# Sensor-Based Air-Gap Monitoring of Elevator Brakes via RMS-Envelope-Guided Transient Impact Extraction and RBF-SVM

**DOI:** 10.3390/s26144606

**Published:** 2026-07-20

**Authors:** Shuaishuai Xing, Jinkui Feng, Chao Wang

**Affiliations:** 1 College of Mechanical Engineering, Xinjiang University, Urumqi 830017, China; xss@stu.xju.edu.cn (S.X.); wangchao@xju.edu.cn (C.W.); 2 China Special Equipment Inspection and Research Institute, Beijing 100029, China

**Keywords:** elevator brake, air-gap monitoring, multi-axis vibration, RMS envelope, transient impact extraction, RBF-SVM

## Abstract

Air-gap variation in elevator brakes affects armature actuation, brake-shoe release/contact processes, and transient vibration responses, making it an important indicator for brake condition monitoring. However, long-duration vibration recordings acquired from brake-mounted sensors contain mixed operating stages, which makes it difficult to isolate short action-related impacts. This study develops an engineering-oriented sensor-based framework for elevator brake air-gap monitoring by combining RMS-envelope-guided transient impact extraction with an RBF-SVM classifier. A triaxial accelerometer was mounted on the right-side brake armature, and a fixed y-axis vibration channel was used as the baseline input for feature construction. The short-time RMS envelope was first used to identify operating-state transition boundaries. Brake-release and brake-engagement impact peaks were then localized within the neighborhoods of these boundaries, and fixed-length transient impact samples were extracted. Time-domain, frequency-domain statistical, and band-energy features were constructed to characterize impact intensity, spectral structure, and energy redistribution under different air-gap conditions. Experiments were conducted on an elevator traction-machine brake under six controlled air-gap states from 0.30 mm to 0.80 mm. The results show that brake-release impact features are more sensitive to air-gap variation than brake-engagement or combined impact features. Using brake-release features, the proposed RMS-IE-SVM method achieved an accuracy of 89.77% and a Macro F1 of 89.85% under the last-file split setting, and a mean accuracy of 83.62% and a mean Macro F1 of 79.07% under file-grouped cross-validation. In the common-sample multi-axis comparison, X + Y + Z feature-level fusion achieved a file-grouped cross-validation accuracy of 89.63% and a Macro F1 of 85.94%. Feature-group ablation shows that time-domain features provide the dominant information, while band-energy features offer complementary information. These results indicate that RMS-envelope-guided transient impact extraction provides an interpretable framework for controlled-condition elevator brake air-gap identification and that multi-axis vibration information can further improve cross-file generalization.

## 1. Introduction

The elevator brake is a critical actuator in the safety protection system of traction elevators, and its operational reliability is directly related to the braking capability of the car and the overall operational safety of the elevator system [1,2]. During long-term service, the brake is affected by factors such as mechanical wear, variations in the air gap, fluctuations in spring preload, and changes in the electromagnetic attraction state, which may lead to abnormalities during brake shoe release, armature motion, and brake engagement [3,4,5]. For elevator brakes, the air gap is an important parameter affecting the actuation stroke and electromagnetic attraction characteristics [6]. When the brake air gap changes, the contact process between the brake shoe and the brake wheel, as well as the transient impact response of the brake mechanism, also changes accordingly. Therefore, identifying the air-gap state of elevator brakes based on vibration signals is of practical engineering significance for condition monitoring and early anomaly warning of elevator brakes.

Recent studies have shown that data-driven methods can be used for intelligent diagnosis of elevator brakes, elevator door systems, and brake-type actuators using measurable signals [1,7,8]. These studies demonstrate the feasibility of signal-based diagnosis for elevator and braking systems. However, most existing work focuses on fault category identification, label-noise handling, or complex model construction, while limited attention has been paid to local action-induced impact responses caused by brake air-gap variation.

Vibration signals can reflect the dynamic changes of mechanical structures during impact, contact, friction, and looseness processes and have been widely used for condition identification and fault diagnosis of mechanical equipment [9,10,11,12,13,14]. In their review of vibration-based condition monitoring, Caesarendra and Tjahjowidodo [15] systematically summarized the roles of time-domain, frequency-domain, and time–frequency-domain features in characterizing degradation trends. Gupta and Pradhan [16] also pointed out that defects in mechanical components, such as rolling bearings, are usually manifested in the time-domain, frequency-domain, or time–frequency-domain features of vibration signals. Li et al. [17] further proposed statistical feature extraction and evaluation methods for fault diagnosis of rotating machinery, indicating that properly constructed statistical features still offer good interpretability in engineering diagnosis. The studies by Zhu et al. [18] and Xie et al. [19] also demonstrated that multi-scale or multi-domain feature fusion can enhance fault feature representation. For elevator brakes, brake release and brake engagement exhibit distinct transient impact characteristics. Therefore, extracting time-domain, frequency-domain statistical features and band-energy features from local impact samples can provide a physically meaningful feature basis for identifying the air-gap state of elevator brakes.

In terms of classification modeling, traditional machine learning methods, especially support vector machines (SVMs), remain suitable for engineering scenarios with limited sample sizes, high-dimensional features, and a need for interpretable diagnostic evidence [20,21,22]. In their review of intelligent fault diagnosis for rotating machinery, Liu et al. [23] pointed out that methods such as K-nearest neighbors (KNN), SVMs, artificial neural networks, and deep learning have been widely applied to mechanical fault identification, among which SVMs still have practical value in small-sample and nonlinear classification tasks. Guo et al. [24] combined the Hilbert envelope spectrum with SVM to classify bearing faults, demonstrating that the integration of envelope information and SVM is suitable for processing vibration signals with impact modulation characteristics. Gryllias and Antoniadis [25] trained an SVM using features derived from a physical model for bearing fault detection in industrial environments, while Islam and Kim [26] fused heterogeneous features and adopted a multi-class SVM to diagnose compound bearing faults. These studies indicate that, if the extracted features can effectively reflect the fault mechanism, SVM-based methods can still achieve stable recognition performance under limited-sample conditions.

Deep learning has been widely used in mechanical fault diagnosis because of its ability to learn hierarchical representations from raw or minimally processed signals [23,27,28,29]. In addition to conventional deep networks, recent studies have explored light-weight neural networks, transfer learning, domain adaptation, foundation-model-based classifiers, and few-shot/meta-learning strategies for machinery fault diagnosis and limited-data classification. For example, compact CNN-based models can directly learn representations from raw or minimally processed vibration signals, while transfer-learning and domain-adaptation methods aim to reuse knowledge learned from related operating conditions or mechanical systems [30,31,32,33,34]. More recently, few-shot meta-learning and tabular foundation models have also provided useful ideas for improving generalization when labeled samples are limited [35,36]. These studies indicate that modern deep learning methods are promising for small-sample mechanical diagnosis. However, for the elevator brake air-gap monitoring task considered in this study, the available data were collected from a single traction machine under controlled air-gap settings, and the physical correspondence between local brake-action impacts and air-gap variation is particularly important. Therefore, this study adopts an interpretable feature-based framework tailored to controlled experimental data and physically meaningful brake-action impacts. This framework provides a physically interpretable baseline for elevator brake air-gap identification under limited controlled experimental data, while deep learning-based extensions remain a promising direction for future cross-equipment and multi-condition studies.

From the perspective of elevator brake condition monitoring, the key problem is not only to classify different brake states but also to obtain action-specific information that is physically related to brake release and brake engagement. Existing elevator-brake-related studies have mainly focused on fault category identification, motion modeling, brake performance testing, or global signal-based diagnosis. These studies provide useful evidence for the feasibility of sensor-based brake monitoring, but the local transient impact responses caused by controlled air-gap variation have not been sufficiently investigated. In long-duration vibration recordings, the stationary stage, brake-release impact, stable operation, brake-engagement impact, and return-to-stationary stage are mixed within the same signal. Directly extracting features from the entire recording may introduce background vibration unrelated to the target brake action, while direct global peak detection may confuse action-related impacts with random impacts during stable operation. Therefore, there remains a need for an interpretable extraction strategy that can locate brake-action-related transient impacts from long-duration vibration records and establish a clearer correspondence between the extracted sensor features and air-gap variation.

To this end, this study proposes a sensor-based elevator brake air-gap monitoring method based on RMS-envelope-guided transient impact extraction and an RBF-SVM classifier, referred to as RMS-IE-SVM. The proposed method first uses the short-time RMS envelope to identify the operating-state transition boundaries of the brake. Then, brake-release and brake-engagement impact peaks are located within the neighborhoods of these boundaries, and fixed-length local impact samples are segmented. Subsequently, time-domain, frequency-domain statistical, and band-energy features are extracted from the impact samples to construct a multi-domain feature set. Finally, a radial basis function kernel support vector machine is employed to identify different brake air-gap states. This method mainly addresses the difficulties in accurately locating target impacts in long-duration vibration signals, reducing the interference of non-target operating vibrations on diagnostic features, and improving the interpretability of vibration-sensor features for air-gap monitoring. It should be noted that this study does not directly conduct long-term brake shoe wear tests. Instead, different geometric clearance states are constructed by adjusting the working air gap of the brake to represent, under controlled conditions, changes in actuation stroke that may be associated with brake shoe wear, mechanism adjustment deviations, or long-term service. This experimental design enables the influence of air-gap variation on the transient impact responses during brake release and brake engagement to be analyzed under controlled conditions. However, the conclusions are mainly applicable to brake air-gap condition identification and equivalent clearance variation diagnosis and should not be directly generalized to all real wear mechanisms.

The main contributions of this study are summarized as follows:

(1) An engineering-oriented vibration-sensor signal processing framework is proposed for elevator brake air-gap monitoring. The framework links long-duration brake-mounted vibration recordings to local brake-action-related transient samples, thereby improving the correspondence between sensor-derived features and the actual brake actuation process.
(2) An RMS-envelope-guided transient impact extraction strategy is developed to identify operating-state transition boundaries and restrict impact peak searching to the neighborhoods of brake-release and brake-engagement transitions. This strategy reduces the interference of non-target vibration stages and provides action-specific impact samples for subsequent feature construction.
(3) Interpretable multi-domain impact features are constructed from the extracted brake-action samples, and cross-file validation is used to evaluate their sensitivity to controlled air-gap variation. Ordered-class metrics, RMS-parameter sensitivity analysis, and single-axis/multi-axis input comparison are further used to assess the robustness and physical interpretability of the proposed framework.

## 2. Methods

### 2.1. Framework of the RMS-IE-SVM Method

**Figure 1:**
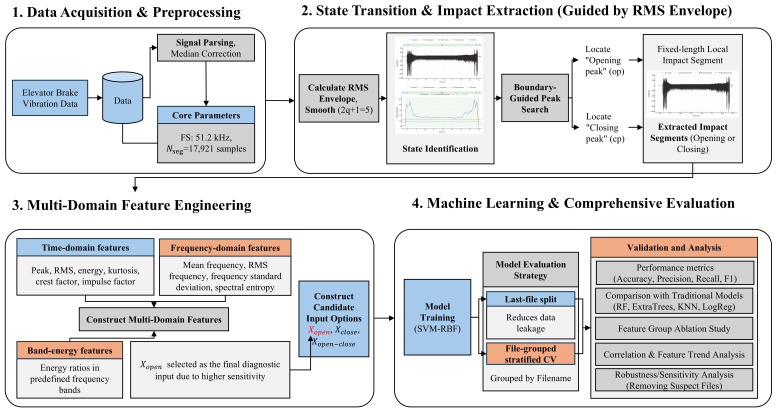
Overall framework of the RMS-IE-SVM method.

The proposed RMS-IE-SVM method extracts action-specific transient impact information from long-duration vibration signals acquired by brake-mounted sensors. It first identifies operating-state transition boundaries using the short-time RMS envelope, then restricts impact peak search to the neighborhoods of these boundaries to obtain brake-release and brake-engagement impact samples. Multi-domain features are subsequently constructed from the extracted samples and fed into an RBF-SVM classifier for air-gap monitoring. Compared with full-signal feature extraction or global peak detection, this strategy improves the correspondence between sensor-derived features and actual brake actuation processes. Figure 1 shows the overall framework of the RMS-IE-SVM method.

### 2.2. RMS-Envelope-Guided Impact Extraction

The RMS value reflects the vibration energy level within a local time window [37,38,39]. For the elevator brake signals investigated in this study, the RMS value remains relatively low during the stationary stage, increases significantly when the brake is released and the system enters the operating stage, and decreases rapidly when the brake is engaged and the system returns to the stationary stage. Therefore, the RMS sequence can be used to describe changes in the operating state of the brake.

Let the vibration signal collected from the elevator brake be expressed as

(1) x[n],n=0,1,…,N−1

where x[n] denotes the vibration amplitude at the n-th sampling point, and N is the total length of the signal. In this study, the sampling frequency is $f_{s}=51.2\;\mathrm{kHz}$.

To reduce the influence of sensor zero-offset bias and low-frequency drift on subsequent analysis, the signal is first median-centered as follows:

(2) $$\tilde{x}[n]=x[n]-\mathrm{median}(x[n])$$

where $\tilde{x}[n]$ denotes the median-centered vibration signal, and median(x[n]) represents the median amplitude of the entire signal. After median centering, the signal amplitude mainly reflects the dynamic vibration changes during brake actuation and operation.

The median-centered signal is divided into short-time blocks with a length of M. The k-th short-time block is expressed as

(3) $$x_{k}=[\tilde{x}[kM],\tilde{x}[kM+1],\ldots ,\tilde{x}[kM+M-1]]$$

where $x_{k}$ denotes the k-th short-time signal block, k is the block index, and M is the number of sampling points in each short-time block. In this study, the RMS block duration is set to 0.05 s; thus, $M=0.05f_{s}=2560$.

The RMS value of the k-th short-time block is defined as

(4) $$r_{k}=\sqrt{\frac{1}{M}\sum_{m=0}^{M-1}{\tilde{x}}^{2}[kM+m]}$$

where $r_{k}$ denotes the RMS value of the k-th short-time block, and m is the sample index within the block.

To reduce the influence of local random impacts during brake release and brake engagement of the elevator traction machine on state identification, a moving average is applied to smooth the RMS sequence:

(5) $${\bar{r}}_{k}=\frac{1}{2q+1}\sum_{i=-q}^{q}r_{k+i}$$

where ${\bar{r}}_{k}$ denotes the smoothed RMS envelope, q is the half-width of the smoothing window, and 2q+1 represents the number of short-time blocks included in the averaging operation. In this study, 2q+1=5, namely q=2. This smoothing operation reduces local random fluctuations while preserving the state-transition trend of the brake.

Considering that multiple data files may be included during data acquisition and that different files may exhibit amplitude differences, an adaptive threshold is constructed using the percentiles of the RMS envelope:

(6) $$r_{\mathrm{low}}=P_{20}({\bar{r}}_{k}),\;\;\;\;r_{\mathrm{high}}=P_{80}({\bar{r}}_{k})$$

where $r_{\mathrm{low}}$ and $r_{\mathrm{high}}$ denote the low-percentile and high-percentile levels of the smoothed RMS envelope, respectively, and $P_{20}(\cdot )$ and $P_{80}(\cdot )$ represent the 20th and 80th percentiles, respectively. This design reduces the influence of overall vibration amplitude differences among different files on threshold determination.

To accurately identify the brake-release transition and brake-engagement transition of the elevator brake, the entering-operation threshold and exiting-operation threshold are defined as

(7) $$T_{\mathrm{on}}=r_{\mathrm{low}}+\alpha_{\mathrm{on}}(r_{\mathrm{high}}-r_{\mathrm{low}}),\;\;\;\;T_{\mathrm{off}}=r_{\mathrm{low}}+\alpha_{\mathrm{off}}(r_{\mathrm{high}}-r_{\mathrm{low}})$$

where $\alpha_{\mathrm{on}}$ and $\alpha_{\mathrm{off}}$ are threshold proportional coefficients satisfying $\alpha_{\mathrm{on}}>\alpha_{\mathrm{off}}$. $T_{\mathrm{on}}$ is used to identify the brake-release transition process from the stationary state to the operating state, whereas $T_{\mathrm{off}}$ is used to identify the brake-engagement transition process from the operating state back to the stationary state. In this study, $\alpha_{\mathrm{on}}=0.45$ and $\alpha_{\mathrm{off}}=0.25$. The parameters q, $\alpha_{\mathrm{on}}$, and $\alpha_{\mathrm{off}}$ were selected according to the amplitude separation between the stationary and operating stages in the RMS envelope. The smoothing half-width q controls the suppression of short random fluctuations, whereas $\alpha_{\mathrm{on}}$ and $\alpha_{\mathrm{off}}$ determine the hysteresis range for entering and exiting the operating state. A relatively higher $\alpha_{\mathrm{on}}$ helps avoid false triggering caused by small stationary-stage fluctuations, while a lower $\alpha_{\mathrm{off}}$ prevents repeated switching around the operating-state boundary. To verify that the extraction results were not overly dependent on a single empirical setting, a local parameter sensitivity analysis was further conducted in the Results section by varying q, $\alpha_{\mathrm{on}}$, and $\alpha_{\mathrm{off}}$ around the adopted values.

Based on the dual-threshold strategy, the operating-state sequence is defined as

(8) $$s_{k}=\left\{\begin{array}{ll} 1, & s_{k-1}=0\;\mathrm{and}\;{\bar{r}}_{k}\geq T_{\mathrm{on}},\\ 0, & s_{k-1}=1\;\mathrm{and}\;{\bar{r}}_{k}\leq T_{\mathrm{off}},\\ s_{k-1}, & \mathrm{otherwise}. \end{array}\right.$$

where $s_{k}$ denotes the operating state of the k-th short-time block. $s_{k}=1$ indicates the operating state, whereas $s_{k}=0$ indicates the stationary state. This dual-threshold hysteresis strategy avoids frequent state transitions when the RMS envelope fluctuates around the threshold.

To identify the state-transition boundaries, the operating-state sequence is differenced as follows:

(9) $$d_{k}=s_{k}-s_{k-1}$$

where $d_{k}$ denotes the state variation at the k-th short-time block. When $d_{k}=1$, the signal changes from the stationary state to the operating state, corresponding to the brake-release transition region. When $d_{k}=-1$, the signal changes from the operating state to the stationary state, corresponding to the brake-engagement transition region.

The sampling point corresponding to the state-transition boundary is given by

(10) $$b_{k}=kM$$

where $b_{k}$ denotes the sampling point position in the raw signal corresponding to the k-th state-transition block. Since the RMS envelope is calculated at the block level, the state boundary is first obtained at the block level and then mapped back to the sampling-point position of the raw signal.

After obtaining the state-transition boundaries, local maximum impact peaks are searched within the neighborhoods of these boundaries:

(11) $$p_{j}=\arg\underset{n\in \left[b_{j}-L_{1},b_{j}+L_{2}\right]}{\max}\left|\tilde{x}\left[n\right]\right|$$

where $p_{j}$ denotes the impact peak position corresponding to the j-th state-transition boundary, $b_{j}$ is the position of the j-th state-transition boundary, and $L_{1}$ and $L_{2}$ denote the search lengths before and after the boundary, respectively. In this study, $L_{1}=L_{2}=0.60f_{s}=30720$. Since brake-release and brake-engagement impacts may appear as either positive or negative spikes, the absolute value of the median-centered signal, $\left|\tilde{x}\left[n\right]\right|$, is used during local peak searching to avoid missing large-amplitude negative impacts. Unlike global peak detection, the proposed method restricts the search range to the neighborhoods of state transitions, thereby reducing the interference of random impacts during the stable operating stage on peak localization.

To ensure cycle consistency between brake-release and brake-engagement samples, each brake-release peak is paired with the nearest subsequent brake-engagement peak:

(12) $$\left(p_{i}^{open},p_{i}^{close}\right)=\left(p_{i}^{open},\min\{p^{close}|p^{close}>p_{i}^{open}\}\right)$$

where $p_{i}^{open}$ denotes the position of the i-th brake-release impact peak, and $p_{i}^{close}$ denotes the position of the matched brake-engagement impact peak. If a brake-release event exists at the end of a file but lacks a corresponding brake-engagement event, the incomplete cycle is discarded.

Taking the impact peak $p_{j}$ as the center, a fixed-length local impact sample is segmented as follows:

(13) $$u_{j}=\left[\tilde{x}\left[p_{j}-N_{pre}\right],\ldots ,\tilde{x}\left[p_{j}\right],\ldots ,\tilde{x}\left[p_{j}+N_{post}\right]\right]$$

where $u_{j}$ denotes the j-th local impact-sample vector, $N_{pre}$ is the number of sampling points retained before the peak, and $N_{post}$ is the number of sampling points retained after the peak. In this study, $N_{\mathrm{pre}}=0.05f_{s}=2560$ and $N_{\mathrm{post}}=0.30f_{s}=15360$. Therefore, the length of each impact sample is

(14) $$N_{seg}=N_{pre}+N_{post}+1=17921$$

The extracted impact sample represents the local transient response of brake release or brake engagement, rather than a complete long-cycle signal covering the entire “stationary–brake release–operation–brake engagement–stationary” process.

### 2.3. Multi-Domain Impact Feature Construction and RBF-Kernel SVM-Based Air-Gap Identification

The j-th local impact sample obtained from Equation (13) is denoted as

(15) $$u_{j}=\left[u_{j}[0],u_{j}[1],\ldots ,u_{j}[N_{\mathrm{seg}}-1]\right]$$

where $u_{j}\left[n\right]$ denotes the vibration amplitude of the n-th sampling point in the j-th impact sample, and $N_{\mathrm{seg}}$ is the length of the impact sample. In this study, features are constructed from three perspectives, namely time domain, frequency-domain statistics, and band energy, to characterize the influence of brake air-gap variation on impact intensity, spectral structure, and energy distribution.

The impact peak is used to describe the maximum transient amplitude during the local impact process:

(16) $$A_{peak}=\underset{n}{\max}\left|u_{j}\left[n\right]\right|$$

where $A_{peak}$ denotes the peak amplitude of the j-th impact sample, and $\left|u_{j}\left[n\right]\right|$ represents the absolute value of the vibration amplitude at the n-th sampling point. This index reflects the maximum local impact intensity during brake release or brake engagement.

The RMS value of the impact sample is used to describe the average vibration intensity within the entire local impact window:

(17) $$A_{rms}=\sqrt{\frac{1}{N_{seg}}\overset{N_{seg}-1}{\underset{n=0}{\sum }}u_{j}^{2}\left[n\right]}$$

where $A_{rms}$ denotes the RMS value of the j-th impact sample, and $u_{j}\left[n\right]$ is the amplitude of the n-th sampling point in the impact sample. Compared with a single-point peak value, the RMS value better reflects the overall vibration level of the local impact process. Variation in the brake air gap may alter the armature actuation stroke and brake shoe release process, thereby causing changes in the RMS value of the brake-release impact.

The impact energy is defined as

(18) $$E=\overset{N_{seg}-1}{\underset{n=0}{\sum }}u_{j}^{2}\left[n\right]$$

where E denotes the total energy of the j-th impact sample. This index describes the accumulated vibration energy in the local transient response during brake release or brake engagement. When the impact sample length is fixed, both energy and RMS can reflect impact intensity, whereas the energy feature places greater emphasis on the cumulative effect of the local response.

To reduce the influence of the excessively large numerical range of the energy feature on model training, logarithmic energy is further constructed as

(19) $$E_{\log}=\log\left(E+\varepsilon \right)$$

where $E_{\log}$ denotes the logarithmic energy of the impact sample, and ε is a small constant introduced to avoid numerical instability when E=0. The logarithmic transformation compresses the dynamic range of the energy feature, making it more suitable for joint input into the classification model together with other features.

Kurtosis is defined as

(20) $$K=\frac{1}{N_{\mathrm{seg}}}\sum_{n=0}^{N_{\mathrm{seg}}-1}{\left(\frac{u_{j}[n]-\mu_{j}}{\sigma_{j}+\varepsilon }\right)}^{4}$$

where K denotes the kurtosis of the j-th impact sample, $\mu_{j}$ and $\sigma_{j}$ denote the mean and standard deviation of the impact sample, respectively, and ε is a small constant introduced to avoid division by zero. A larger kurtosis value indicates more prominent impulsive components in the signal. During brake release or brake engagement, local collision or release impacts may lead to an increase in kurtosis.

The crest factor is defined as

(21) $$C_{f}=\frac{A_{peak}}{A_{rms}}$$

where $C_{f}$ denotes the crest factor, $A_{peak}$ is the impact peak value, and $A_{rms}$ is the RMS value of the impact sample. A larger crest factor indicates that the local peak is more prominent relative to the overall vibration level.

To analyze the spectral distribution of the impact signal, the local impact sample is first mean-removed and multiplied by a Hann window before frequency-domain analysis to reduce spectral leakage. Then, a real-valued fast Fourier transform is performed on the processed impact sample to obtain the amplitude spectrum and power spectrum. The discrete Fourier transform can be expressed as

(22) $$U_{j}[k]=\sum_{n=0}^{N_{\mathrm{seg}}-1}w[n]\left(u_{j}[n]-\mu_{j}\right)e^{-i2\pi kn/N_{\mathrm{seg}}}$$

where $w\left[n\right]$ is the Hann window function, $U_{j}[k]$ denotes the complex spectrum of the j-th impact sample at the k-th frequency point, k is the frequency index, and i is the imaginary unit. The Fourier transform converts the time-domain impact sample into a frequency-domain representation, which is used to analyze whether air-gap variation changes the frequency components of the impact signal.

The power spectrum of the impact sample at the k-th frequency point is defined as

(23) $$P_{j}\left[k\right]={\left|U_{j}\left[k\right]\right|}^{2}$$

where $P_{j}\left[k\right]$ denotes the spectral energy of the j-th impact sample at frequency point k. The power spectrum is used for subsequent calculation of frequency-band energy ratios and spectral entropy.

The energy ratio of the b-th frequency band is defined as

(24) $$R_{b}=\frac{\sum_{k\in B_{b}}P_{j}\left[k\right]}{\sum_{k}P_{j}\left[k\right]}$$

where $R_{b}$ denotes the energy proportion of the b-th frequency band; $B_{b}$ denotes the set of frequency indices corresponding to the b-th predefined frequency band. In this study, multiple frequency-band energy ratios are used to describe the distribution of impact energy in different frequency ranges. If air-gap variation causes changes in the frequency components of the impact signal, the energy proportions in different frequency bands will also change accordingly.

To describe the complexity of the spectral energy distribution, the power spectrum is first normalized as

(25) $${\hat{P}}_{j}\left[k\right]=\frac{P_{j}\left[k\right]}{\sum_{k}P_{j}\left[k\right]}$$

where ${\hat{P}}_{j}\left[k\right]$ denotes the normalized power spectrum, which reflects the proportion of the energy at the k-th frequency point in the total spectral energy.

Based on the normalized power spectrum, the spectral entropy is defined as

(26) $$H_{s}=-\frac{1}{\log K}\sum_{k=1}^{K}{\hat{P}}_{j}[k]\log\left({\hat{P}}_{j}[k]+\varepsilon \right)$$

where $H_{s}$ denotes the normalized spectral entropy, K is the number of frequency points involved in the calculation, and ε is a small constant introduced to avoid taking the logarithm of zero. In this study, normalized spectral entropy is adopted, so that the value of $H_{s}$ is approximately within the range of 0–1. A larger spectral entropy indicates a more dispersed spectral energy distribution, whereas a smaller spectral entropy indicates that energy is more concentrated in a few frequency components. This feature can be used to describe the influence of brake air-gap variation on the spectral complexity of the impact signal.

Finally, the feature vector of the j-th impact sample is expressed as

(27) $$z_{j}=\left[F_{t},F_{f},F_{b}\right]$$

where $z_{j}$ denotes the feature vector of the j-th impact sample, and $F_{t}$, $F_{f}$, and $F_{b}$ denote the sets of time-domain features, frequency-domain features, and band-energy features, respectively. The multi-domain features jointly describe changes in impact intensity, spectral structure, and band energy redistribution caused by brake air-gap variation.

For each brake-release or brake-engagement impact sample, 33 diagnostic features are finally constructed in this study, including 22 time-domain features, 5 frequency-domain statistical features, and 6 band-energy features. Among them, the time-domain features mainly describe the impact amplitude, energy, distribution pattern, and impulsiveness; the frequency-domain statistical features describe spectral structure information, such as spectral centroid, frequency dispersion, and spectral entropy; and the band-energy features describe the energy proportions in different frequency ranges. It should be noted that open_peak_idx and open_abs_peak_amp are retained only as metadata during the impact extraction process and are not used as diagnostic features for model training or feature-group ablation analysis. For the combined brake-release and brake-engagement input $X_{\mathrm{open}\text{-}\mathrm{close}}$, difference and ratio features between the brake-release features and the brake-engagement features are further constructed, resulting in a 94-dimensional combined feature vector.

To eliminate dimensional differences among different features, feature standardization is performed as follows:

(28) $$z_{ij}^{\prime }=\frac{z_{ij}-\mu_{j}}{\sigma_{j}}$$

where $z_{ij}$ denotes the j-th original feature value of the i-th sample, $z_{ij}^{\prime }$ denotes the standardized feature value, and $\mu_{j}$ and $\sigma_{j}$ denote the mean and standard deviation of the j-th feature calculated from the training set, respectively. The standardization parameters are calculated only from the training set and then applied to the test set to avoid data leakage.

In this study, an RBF-kernel SVM is adopted for air-gap state identification. The RBF kernel function is defined as

(29) $$K\left(z_{i},z_{j}\right)=\exp\left(-\gamma ∥z_{i}-z_{j}{∥}^{2}\right)$$

where $K\left(z_{i},z_{j}\right)$ denotes the similarity between samples $z_{i}$ and $z_{j}$ in the RBF kernel space, γ is the kernel parameter, and $∥z_{i}-z_{j}{∥}^{2}$ denotes the squared Euclidean distance between the two feature vectors. The RBF kernel can construct nonlinear classification boundaries [40,41], making it suitable for handling nonlinear distributions and local overlap of impact features under different air-gap states.

During model training, possible missing values are first imputed using the median values of the training-set features. The features are then standardized using the means and standard deviations calculated from the training set, and the same imputation and standardization parameters are applied to the test set. For file-grouped cross-validation, the median imputer and Z-score scaler were refitted independently within each training fold. The fitted imputation values, feature means, and feature standard deviations were then applied to the corresponding validation fold only. Therefore, no information from the validation or test files was used during feature imputation, standardization, or model training. In this study, the parameters of the RBF-kernel SVM classifier are set as C=10 and γ=scale, and class_weight = balanced is adopted to reduce the influence of slight class imbalance on the classification results. For each evaluation setting, all compared models used the same data partitioning strategy to ensure a fair comparison.

The final predicted class is determined by the maximum discriminant function:

(30) $$\hat{y}=\underset{c}{argmax}\;f_{c}\left(z\right)$$

where $\hat{y}$ denotes the predicted brake air-gap class, c denotes the candidate class index, and $f_{c}\left(z\right)$ denotes the discriminant function indicating that sample z belongs to class c. The brake air-gap states considered in this study include six classes: 0.30 mm, 0.40 mm, 0.50 mm, 0.60 mm, 0.70 mm, and 0.80 mm.

## 3. Experimental Setup and Vibration Data

### 3.1. Structure and Working Process of the Elevator Traction Machine Brake

**Figure 2:**
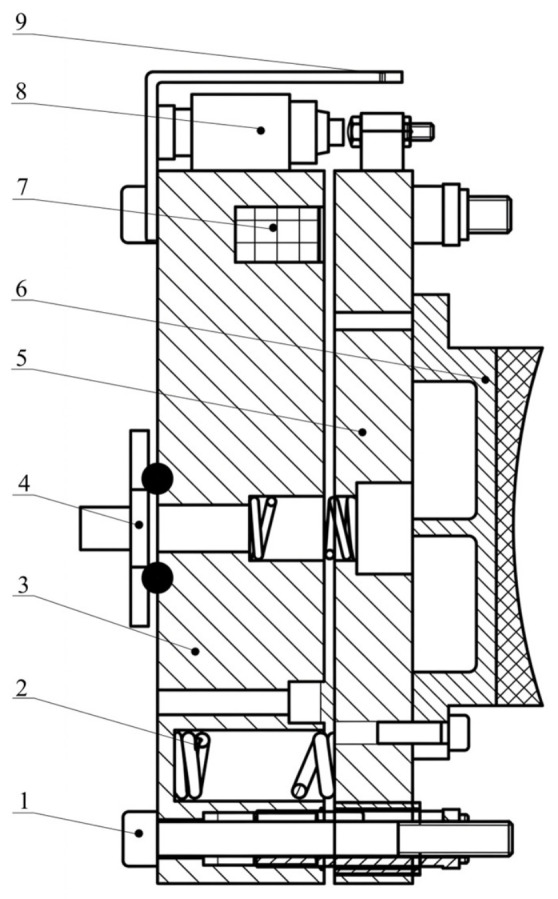
Structure of the brake: 1. mounting screw; 2. brake spring; 3. electromagnet; 4. manual brake release device; 5. armature; 6. brake block; 7. excitation coil; 8. microswitch; 9. protective cover.

The block brake of the elevator traction machine mainly consists of mounting screws, brake springs, an electromagnet, a manual brake release device, an armature, brake blocks, an excitation coil, a microswitch, and a protective cover, as shown in Figure 2. The block brake is directly mounted on the traction machine base and has two operating states: brake-block closing and brake-block opening. Its working principle is as follows. When excitation coil 7 is energized, armature 5 is attracted by electromagnet 3, the brake shoes of brake block 6 are released, and braking is disengaged. When excitation coil 7 is de-energized, armature 5 moves under the thrust of brake spring 2, causing the brake shoes of brake block 6 to brake the traction sheave hub.

### 3.2. Experimental Data Acquisition

#### 3.2.1. Experimental Platform

**Figure 3:**
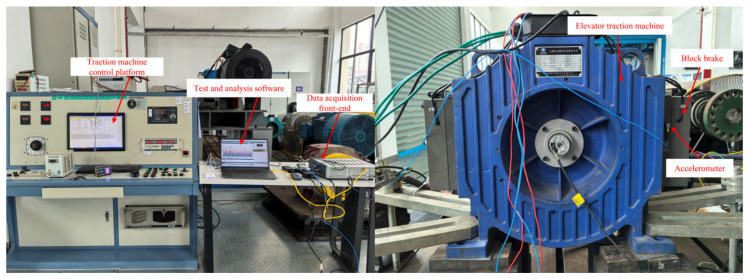
Vibration signal test platform for the traction machine. The Chinese text visible on the traction machine is the original manufacturer nameplate information.

In this study, a vibration signal test platform for the traction machine brake was established using a Shenyang Blue-Light gearless permanent-magnet synchronous elevator traction machine as the experimental object. The experimental system mainly consisted of an elevator traction machine, a block brake, a triaxial accelerometer, a traction machine control platform, a data acquisition front end, and test analysis software, as shown in Figure 3.

During the test, a PCB 356A33 triaxial accelerometer was mounted on the outer surface of the right-side armature of the brake, as shown in Figure 3, close to the electromagnetic attraction surface to collect vibration response signals during brake release, stable operation, and brake engagement. The sensor was fixed using adhesive mounting, and the measurement directions included the x, y, and z directions. The raw voltage signal in the y direction was used as the baseline input for modeling to establish a simple and consistent single-channel monitoring framework. For this y-axis baseline analysis, all features were calculated from the same acquisition channel and the same signal unit; therefore, the signals under different air-gap conditions were comparable. Considering the possible directional sensitivity of brake impact responses, the *x*-axis, *z*-axis, vector magnitude, and X + Y + Z feature-level fusion inputs were also considered in the multi-axis comparison.

The acquired signals were transmitted to the computer through a Siemens LMS SCADAS Mobile data acquisition front end, and signal acquisition control, real-time monitoring, and data storage were performed using Siemens LMS Test.Lab software (Simcenter Testlab 2306). To ensure the comparability of data under different air-gap states, the sensor installation position, installation direction, fastening method, and sampling parameters were kept consistent throughout the experiment. This installation position was selected to enhance the sensitivity of the measured vibration signal to armature actuation and brake-release/brake-engagement impacts, thereby improving the physical relevance of the extracted transient features. The experimental equipment and its main technical parameters are listed in Table 1.

#### 3.2.2. Data Acquisition

During service, brake shoe wear, mechanism adjustment deviations, and spring preload variation may change the geometric clearance and effective actuation stroke of the brake. To obtain controllable equivalent clearance states, this study adjusted the brake air gap and analyzed its influence on transient vibration responses during brake release and engagement. Because long-term wear tests were not conducted, the adjusted air-gap states were used to represent controlled clearance variation rather than all real wear mechanisms.

Different air-gap states were obtained by adjusting the brake mounting screws and were measured using a feeler gauge with a resolution of 0.008 mm. The brake air gap referred to in this study is defined as the working air gap between the armature and the electromagnetic attraction surface. The air gap was measured in the de-energized brake-engaged state. Six positions, namely the upper, middle, and lower points on both the front and rear sides of the right brake arm, were measured, and their average was used as the air-gap value for the corresponding condition. For each nominal air-gap state, the brake was first adjusted to the target clearance by the mounting screws in the de-energized brake-engaged state. After the adjustment, the six-point feeler-gauge measurement was repeated to confirm that the measured average value was consistent with the nominal air-gap state. During data acquisition, the brake structure was not further adjusted, and the same sensor position, mounting direction, adhesive fixation, sampling frequency, running speed, running direction, no-load condition, and brake coil voltage were maintained for all air-gap states. This procedure was used to improve the repeatability of the controlled air-gap states and to reduce the influence of non-gap operating factors on the vibration features. It should be noted that the six fixed measurement points on the front and rear sides of the right brake arm were used to characterize the air gap mainly to ensure consistency in measurement positions among different operating conditions. For issues such as left–right brake arm air-gap asymmetry and multi-point air-gap distribution of the whole brake, a bilateral multi-point measurement scheme can be further extended in future research.

The specified air-gap range of the SDZK-8500 brake is 0.30–0.40 mm. Preliminary experiments showed that the brake could not engage normally when the air gap increased to 0.80 mm. Therefore, six air-gap states were set in this study, namely 0.30 mm, 0.40 mm, 0.50 mm, 0.60 mm, 0.70 mm, and 0.80 mm. The sampling frequency was set to 51.2 kHz, corresponding to a Nyquist frequency of 25.6 kHz, which can cover the high-frequency vibration components in brake-release and brake-engagement impacts. To reduce the influence of variations in operating conditions on the vibration features, the same operating condition was maintained for all air-gap states. The experimental running speed was 168 r/min, the running direction was forward rotation, the load condition was no-load, and the brake coil supply voltage was 110 V. Each acquisition file contained multiple complete brake-release–brake-engagement action processes. A typical raw vibration signal included the stages of stationary state, brake-release impact, stable operation, brake-engagement impact, and stationary state again.

#### 3.2.3. Dataset Construction

Based on the RMS-envelope-guided impact extraction method proposed in Section 2, complete brake-release–brake-engagement cycles were extracted from the raw vibration signals. A total of 985 complete cycle samples were obtained, including 985 brake-release impact samples and 985 brake-engagement impact samples. Each local impact sample had a length of 17,921 sampling points, corresponding to a sampling duration of approximately 0.35 s. The numbers of brake-release samples under different air-gap states were 159, 160, 169, 171, 173, and 153, respectively, and the corresponding numbers of acquisition files were 12, 11, 15, 13, 13, and 12, respectively.

To evaluate the generalization ability of the model across different acquisition files, a last-file split was adopted as the main hold-out testing strategy in this study. Specifically, the last acquisition file in each air-gap class was selected as the test file, while the remaining files were used as training files. Under this setting, the training set contained 897 brake-release impact samples, and the test set contained 88 brake-release impact samples. The numbers of test samples for the six air-gap classes were 14, 14, 15, 15, 15, and 15, respectively. The sample distribution is shown in Table 2. In addition to the last-file split, file-grouped cross-validation was further adopted for cross-file validation to ensure that samples from the same acquisition file did not simultaneously appear in the training and test sets, thereby reducing the risk of data leakage caused by adjacent cycle samples from the same file.

## 4. Results and Discussion

### 4.1. Validation of RMS-Envelope-Based Impact Extraction Results

**Figure 4:**
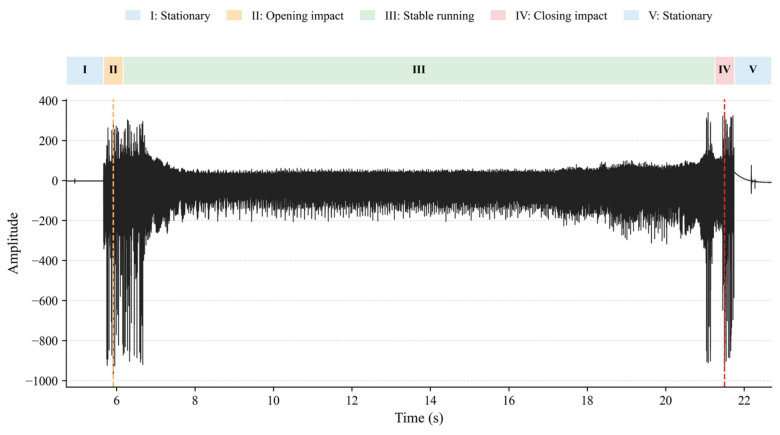
Raw vibration signal of the elevator brake and operating-stage segmentation; the vertical dashed lines indicate the boundaries between adjacent operating stages.

The raw data simultaneously contain the stationary stage, brake-release impact, stable operating stage, and brake-engagement impact. If features are extracted directly from the raw signal, background information from the stationary and stable operating stages will be introduced, thereby weakening the local impact information related to the air gap. Therefore, the operating stages of the data are first segmented in this study, as shown in Figure 4.

**Figure 5:**
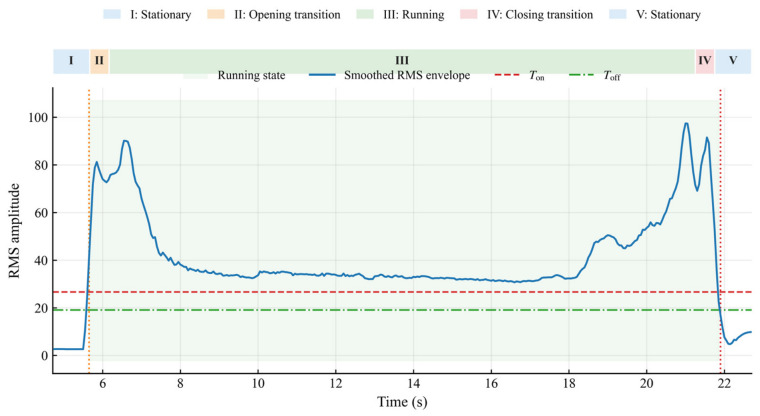
Operating-state identification and state-transition boundary detection based on the RMS envelope; the vertical dotted lines indicate the detected brake-release and brake-engagement transition boundaries.

The RMS envelope can effectively identify the motion state of the brake. Based on the principle that the RMS-envelope amplitude is relatively low during the stationary stage and increases after brake release, the transition of the brake into the operating state can be effectively identified. Similarly, based on the principle that the RMS envelope decreases after brake engagement, the return of the brake to the stationary state can also be effectively identified. Figure 5 shows the operating-state identification results based on the RMS envelope. These results indicate that the short-time RMS envelope can effectively reflect the state-transition process of the brake from the stationary state to the operating state and from the operating state back to the stationary state.

**Figure 6:**
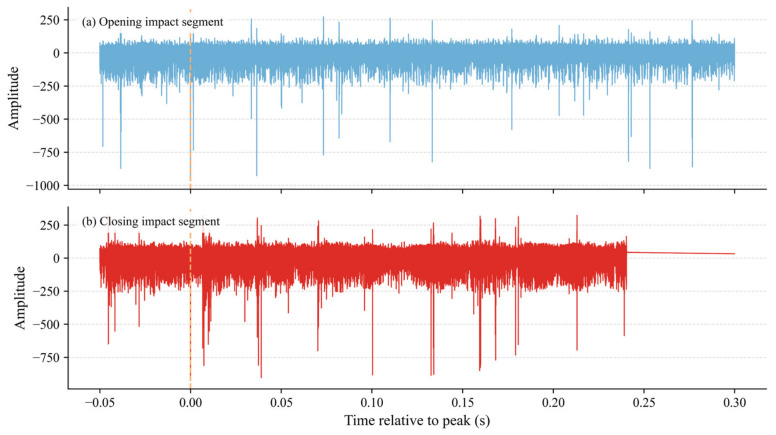
Extracted local impact samples of brake release and brake engagement.

During sample extraction, not only the impact peak itself but also the short pre-peak transition and post-peak decay response are included. Therefore, the extracted samples represent the local transient process of brake release or brake engagement, rather than a complete long-cycle signal. Figure 6 shows the extracted local impact samples of brake release and brake engagement.

**Figure 7:**
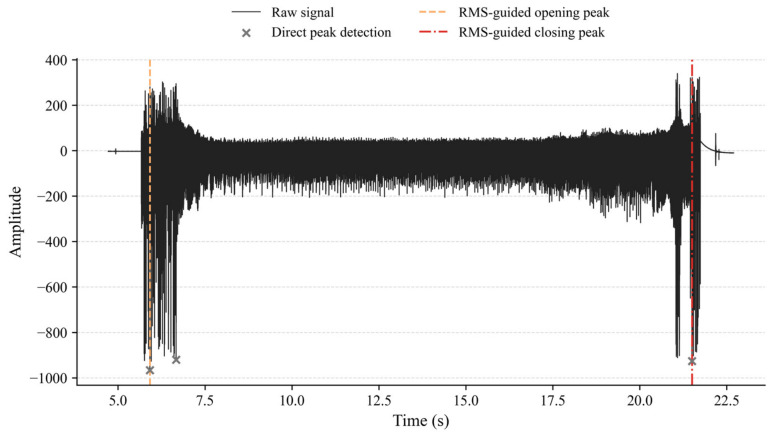
Comparison between RMS-envelope-guided impact extraction and direct peak detection results.

In the peak detection process, direct peak detection may identify additional peaks during the stable operating stage, whereas the proposed method restricts the peak search to the vicinity of the state-transition boundaries. Therefore, the extracted peaks are more consistent with the actual brake-release and brake-engagement processes of the brake. As shown in Figure 7, direct peak detection identifies multiple peaks during brake release, resulting in false detections, whereas the RMS-envelope-guided impact extraction method can accurately locate the brake-release and brake-engagement peaks. These results indicate that the proposed extraction strategy transforms long-duration sensor recordings into action-specific transient samples, which provides a more targeted feature basis for air-gap monitoring than direct analysis of the complete vibration signal.

To evaluate whether the extraction results were overly dependent on the selected RMS-envelope parameters, a local sensitivity analysis was conducted by varying the smoothing half-width q, the entering-operation coefficient $\alpha_{\mathrm{on}}$, and the exiting-operation coefficient $\alpha_{\mathrm{off}}$ around the adopted setting. For each parameter setting, complete brake-release–brake-engagement cycles were extracted again, and the brake-release features were evaluated using the same file-grouped cross-validation strategy. Table 3 shows that all tested parameter settings extracted the same number of complete cycles, namely 985. The file-grouped cross-validation accuracy varied only from 83.42% to 83.72%, and the Macro F1 varied from 78.82% to 79.15%. These results indicate that the RMS-envelope-guided extraction process was stable within the tested local parameter range. Therefore, the adopted setting q=2, $\alpha_{\mathrm{on}}=0.45$, and $\alpha_{\mathrm{off}}=0.25$ was not a unique over-tuned choice but a representative setting within a stable parameter region.

### 4.2. Sensitivity Analysis of Impact Features to Brake Air-Gap Variation

**Figure 8:**
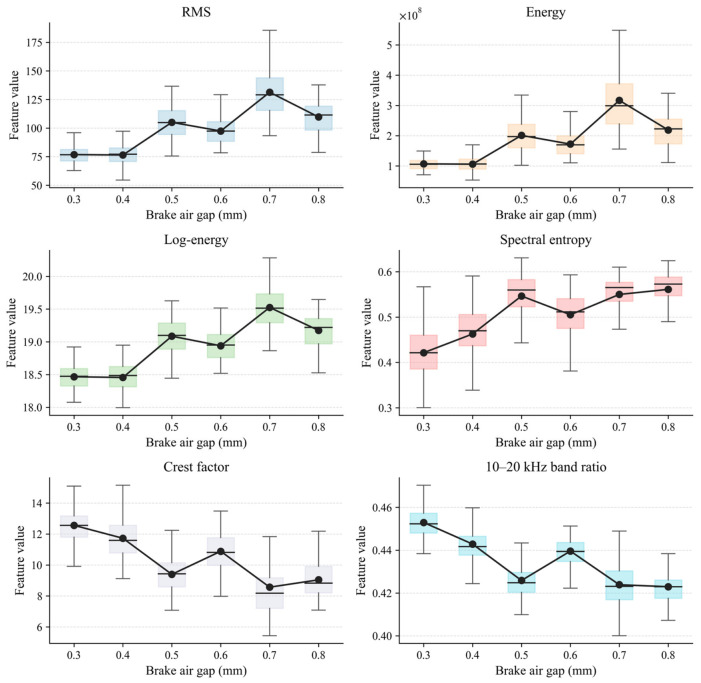
Variation trends of key brake-release impact features under different brake air gaps.

Figure 8 shows the distribution changes of key brake-release impact features under different brake air gaps, including RMS, energy, logarithmic energy, spectral entropy, crest factor, and the 10–20 kHz band energy ratio. It can be observed that RMS, energy, and logarithmic energy generally show an increasing trend with the increase in the air gap, although certain fluctuations occur under the 0.60 mm and 0.80 mm conditions. This indicates that air-gap variation affects the transient impact intensity during the brake-release stage, but this effect is not strictly monotonic. Spectral entropy also shows an overall increasing trend, indicating that air-gap variation changes the complexity of the spectral energy distribution of the impact signal. In contrast, the crest factor and the 10–20 kHz band energy ratio generally show decreasing trends, suggesting that the prominence of local peaks relative to the overall vibration level and the proportion of energy in certain high-frequency components change with air-gap variation.

Correlation analysis further verifies the above observations. As shown in Table 4, RMS, Energy, and Log-energy all exhibit strong positive correlations with the brake air gap, with Spearman correlation coefficients of 0.7333. The feature 10–20 kHz band ratio shows an obvious negative correlation with the air gap, with a Spearman correlation coefficient of −0.6676. The feature Crest factor also exhibits a negative correlation with the air gap, with a Spearman correlation coefficient of −0.6521. In addition, Spectral entropy shows a positive correlation with the air gap, with a Spearman correlation coefficient of 0.6166. These results indicate that brake-release impact intensity, spectral complexity, and band energy distribution can reflect brake air-gap variation from different perspectives.

It should be noted that the correlation coefficients in Table 4 were calculated at the cycle-sample level. Since adjacent cycles extracted from the same acquisition file may not be fully independent, the *p*-values are used only as auxiliary indicators rather than strict evidence under the independent-sample assumption. Therefore, this study emphasizes the direction and magnitude of the Pearson and Spearman coefficients. To further reduce the influence of within-file sample correlation, an additional file-mean-level correlation analysis was conducted by averaging each feature within each acquisition file. The main trends remained consistent at the file level. For example, RMS, energy, and logarithmic energy still showed strong positive Spearman correlations with the air gap, whereas crest factor and the 10–20 kHz band energy ratio still showed strong negative correlations. This confirms that the feature–air-gap associations were not solely caused by repeated adjacent cycles within the same file.

### 4.3. Diagnostic Performance Evaluation, Ordered-Class Metrics, Model Comparison, and Ablation Analysis

In this study, accuracy, precision, recall, and F1-score are used to evaluate model performance. Accuracy is defined as

(31) $$\mathrm{Accuracy}=\frac{N_{\mathrm{correct}}}{N_{\mathrm{total}}}$$

where $N_{\mathrm{correct}}$ denotes the number of correctly classified samples in the test set, and $N_{\mathrm{total}}$ denotes the total number of test samples.

For a given class, precision, recall, and F1-score are defined as

(32) $$\mathrm{Precision}=\frac{TP}{TP+FP},\;\;\;\;\mathrm{Recall}=\frac{TP}{TP+FN},\;\;\;\;F_{1}=\frac{2\cdot \mathrm{Precision}\cdot \mathrm{Recall}}{\mathrm{Precision}+\mathrm{Recall}}$$

where TP denotes the number of samples correctly predicted as this class, FP denotes the number of samples from other classes incorrectly predicted as this class, and FN denotes the number of samples from this class incorrectly predicted as other classes. For the multi-class classification task, both Macro F1 and Weighted F1 are used to evaluate the overall classification performance. Macro F1 is the arithmetic mean of the F1-scores of all classes and reflects the balanced recognition ability of the model across different classes. Weighted F1 is calculated by weighting the F1-score of each class according to the number of samples in that class and reflects the overall classification performance when the class sample sizes are not completely balanced. Since the sample numbers of different air-gap classes in this study are slightly different, Macro F1 and Weighted F1 are reported together with accuracy.

Since the six brake air-gap states have a natural order, additional ordered-class metrics were introduced to evaluate the physical severity of misclassification. The six air-gap states, namely 0.30 mm, 0.40 mm, 0.50 mm, 0.60 mm, 0.70 mm, and 0.80 mm, were encoded as ordinal ranks. In this way, a misclassification between adjacent air-gap levels is penalized less than a misclassification between distant levels. The mean absolute class error, the mean absolute gap error, and the adjacent-level tolerant accuracy were calculated as follows:

(33) $$\mathrm{MACE}=\frac{1}{N}\sum_{i=1}^{N}\left|r_{i}-{\hat{r}}_{i}\right|,\;\;\;\;\mathrm{MAGE}=\frac{1}{N}\sum_{i=1}^{N}\left|g_{i}-{\hat{g}}_{i}\right|$$

(34) $${\mathrm{Acc}}_{\mathrm{adj}}=\frac{1}{N}\sum_{i=1}^{N}I\left(\left|r_{i}-{\hat{r}}_{i}\right|\leq 1\right)\times 100\%$$

where N is the number of test samples, $r_{i}$ and $\hat{r}$ are the true and predicted ordinal ranks of the i-th sample, respectively, and $g_{i}$ and ${\hat{g}}_{i}$ are the corresponding true and predicted air-gap values in millimeters. I(⋅) denotes the indicator function, which equals 1 when the condition is satisfied and 0 otherwise. MACE measures the average class-rank deviation, MAGE measures the average air-gap prediction deviation in millimeters, and ${\mathrm{Acc}}_{\mathrm{adj}}$ measures the proportion of samples whose predicted class is either correct or within one adjacent air-gap level.

**Figure 9:**
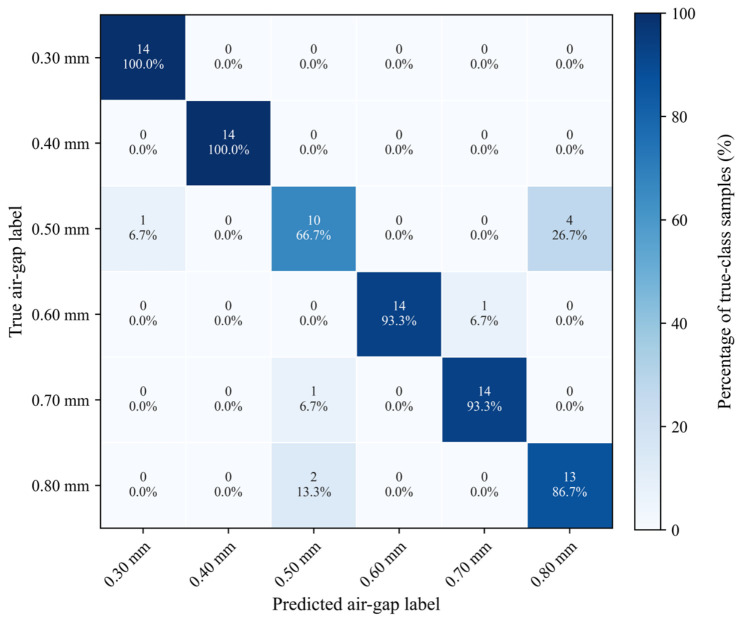
Confusion matrix of RMS-IE-SVM based on brake-release impact features under the last-file split setting. Rows represent true air-gap labels, columns represent predicted air-gap labels, and each cell reports the number of test samples and the corresponding row-normalized percentage.

Figure 9 shows the confusion matrix of RMS-IE-SVM based on brake-release impact features. The test set was constructed using the last-file split strategy, in which the last acquisition file of each air-gap class was selected as the test file, resulting in a total of 88 test samples. As shown in Figure 9, the 0.30 mm and 0.40 mm air-gap states achieve the best recognition performance, with all test samples correctly classified. The 0.60 mm and 0.70 mm states also show high recognition rates. The main misclassifications are concentrated between the 0.50 mm and 0.80 mm classes. Specifically, in the 0.50 mm class, one sample is misclassified as 0.30 mm and four samples are misclassified as 0.80 mm; in the 0.80 mm class, two samples are misclassified as 0.50 mm. The number of correctly classified samples on the diagonal of the confusion matrix is 79, and the total number of test samples is 88, corresponding to an accuracy of 89.77%. This result indicates that brake-release impact features under different air-gap states generally have good separability, although local feature overlap still exists between the 0.50 mm and 0.80 mm classes.

To verify the diagnostic effectiveness of different impact inputs, this study compares the classification performance of three types of inputs, namely $X_{\mathrm{open}}$, $X_{\mathrm{close}}$, and $X_{\mathrm{open}\text{-}\mathrm{close}}$, under traditional machine learning models. The results are shown in Table 5, where each input type is reported under its corresponding optimal model. The results show that the best model for the brake-release impact input $X_{\mathrm{open}}$ is RMS-IE-SVM, with an accuracy of 89.77%, a Macro F1 of 89.85%, and a Weighted F1 of 89.66%. The best model for the brake-engagement impact input $X_{\mathrm{close}}$ is Logistic Regression, with accuracy and Macro F1 of 71.59% and 71.28%, respectively. The best model for the combined brake-release and brake-engagement input $X_{\mathrm{open}\text{-}\mathrm{close}}$ is Random Forest, with accuracy and Macro F1 of 81.82% and 82.51%, respectively. These results indicate that, under the current feature construction and model selection framework, brake-release impact features have stronger representation capability for air-gap states. Therefore, $X_{\mathrm{open}}$ is used as the main diagnostic input in the subsequent analysis.

After determining brake-release impact features as the main input, different traditional machine learning models are further compared under the same $X_{\mathrm{open}}$ input. The results are shown in Table 6. All models were evaluated using the same last-file training/test split to ensure a fair comparison.

Table 6 shows that RMS-IE-SVM achieved the best performance among the compared models, with an accuracy of 89.77% and a Macro F1 of 89.85%. Random Forest, Extra Trees, and Logistic Regression achieved moderate performance, whereas KNN showed the lowest result. This indicates that brake-release impact features exhibit nonlinear distributions and local class overlap, making the RBF-SVM more suitable under the limited-sample condition considered in this study.

Although the fixed *y*-axis signal was used as the baseline input for the single-channel monitoring framework, the brake-release impact response may exhibit direction-dependent vibration characteristics. Therefore, the influence of vibration direction and multi-axis feature fusion was evaluated under two cross-file settings: last-file split and file-grouped cross-validation. Five input types were compared, including *x*-axis features, y-axis features, *z*-axis features, vector magnitude features, and X + Y + Z feature-level fusion features. To ensure a fair comparison, all inputs were evaluated using the same 985 brake-release impact samples from the same 76 acquisition files. For file-grouped cross-validation, identical grouped folds were used for all input types. As shown in Table 7, the diagnostic performance varied among different vibration directions and evaluation settings. Under the last-file split setting, the y-axis input and the X + Y + Z feature-level fusion input achieved similar accuracy values of 89.77%, indicating that the fixed y-axis signal can provide an effective single-channel baseline under this hold-out evaluation setting. However, under the more stringent file-grouped cross-validation setting, the performance difference among input types became more evident. Among the single-axis inputs, the *z*-axis features achieved the highest grouped-CV accuracy and Macro F1, reaching 87.41% and 83.86%, respectively. The vector magnitude input did not outperform the single-axis features, suggesting that direct magnitude synthesis may lose useful directional information. The X + Y + Z feature-level fusion input achieved the best grouped-CV performance, with an accuracy of 89.63%, a Macro F1 of 85.94%, a MAGE of 0.0276 mm, and an adjacent-level tolerant accuracy of 91.44%. Compared with the *y*-axis baseline, feature-level fusion improved the grouped-CV accuracy by 6.01 percentage points and the Macro F1 by 6.87 percentage points. These results indicate that the *y*-axis signal is suitable as a simple single-channel baseline, while multi-axis feature-level fusion can improve cross-file generalization for elevator brake air-gap identification.

**Figure 10:**
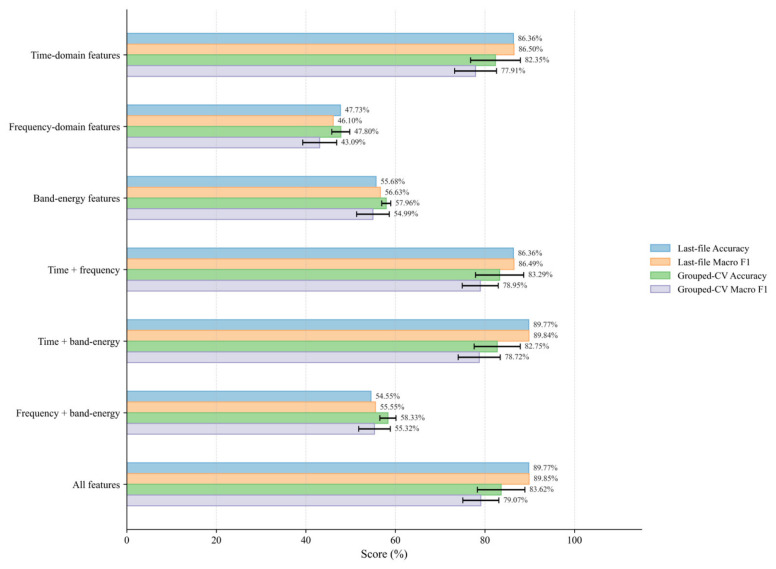
Comparison of the diagnostic performance of RMS-IE-SVM under different feature-group inputs.

To analyze the contributions of different feature groups to brake air-gap identification, this study investigates the diagnostic performance of time-domain features, frequency-domain statistical features, band-energy features, and their combinations. The results are shown in Figure 10. The feature-group ablation experiments use the same RMS-IE-SVM classifier as the main model, namely an RBF-kernel SVM with C=10, γ=scale, and class_weight = balanced. During feature column selection, open_peak_idx and open_abs_peak_amp are retained only as metadata during the impact extraction process and are not used as diagnostic features for model training. Therefore, the final number of features used for brake-release-based air-gap identification is 33.

As shown in Figure 10, among the single feature groups, time-domain features achieve the best performance. Under the last-file split, the accuracy and Macro F1 are 86.36% and 86.50%, respectively; under file-grouped cross-validation, the mean accuracy and mean Macro F1 are 82.35% and 77.91%, respectively. In contrast, frequency-domain statistical features and band-energy features show lower performance when used alone. Specifically, frequency-domain statistical features achieve an accuracy and Macro F1 of 47.73% and 46.10% under the last-file split, whereas band-energy features achieve 55.68% and 56.63%, respectively. This indicates that brake air-gap variation is mainly reflected in the time-domain responses of brake-release impacts, such as amplitude, energy, and impulsiveness, while relying solely on frequency-domain statistical or band-energy features is insufficient for stable discrimination of different air-gap states.

Combined features can further improve recognition performance. The Time + band-energy combination achieves an accuracy of 89.77% and a Macro F1 of 89.84% under the last-file split, which is close to the performance of all-feature input, namely 89.77% and 89.85%. Under file-grouped cross-validation, the all-feature input achieves a mean accuracy of 83.62% and a mean Macro F1 of 79.07%, representing the best overall performance among all feature combinations. These results indicate that time-domain features are the main information source for air-gap identification, while band-energy features provide complementary information to time-domain features. The all-feature input maintains relatively stable recognition performance in cross-file evaluation.

### 4.4. Stability Analysis and Discussion

To evaluate the generalization ability of the model across different acquisition files, file-grouped cross-validation was adopted for cross-file validation in this study. All samples from the same acquisition file were assigned to the same data fold, so as to avoid data leakage caused by adjacent cycle samples from the same file appearing simultaneously in the training and test sets. In addition to the evaluation on the complete dataset, two suspected files, namely 0.5mm15Y.mat and 0.8mm12Y.mat, which exhibited feature overlap in the misclassification analysis, were further removed, and the sensitivity analysis was repeated. These two files were not removed a priori before model training but were identified as boundary samples after misclassification analysis and feature distribution inspection. Therefore, the removal experiment was used only to evaluate the sensitivity of the model to suspected overlapping files, rather than as the main basis for performance evaluation. The results are shown in Table 8.

On the complete dataset, RMS-IE-SVM achieved a mean accuracy of 83.62% and a mean Macro F1 of 79.07% under file-grouped cross-validation. After removing the suspected feature-overlapping files, the mean accuracy under file-grouped cross-validation was 83.65%, and the mean Macro F1 was 80.23%. It can be observed that the average accuracy remained basically stable after removing the suspected files, while the Macro F1 slightly improved, indicating that a small number of suspected overlapping files did not change the overall conclusion regarding the effectiveness of the proposed method.

Meanwhile, under the last-file split setting, the accuracy and Macro F1 of the complete dataset were 89.77% and 89.85%, respectively. After removing the suspected files, the accuracy and Macro F1 were 88.89% and 87.33%, respectively, which were slightly lower than those obtained using the complete dataset. Therefore, the experiment involving the removal of suspected files should be regarded as a sensitivity analysis rather than evidence of main performance improvement. Overall, the file-grouped cross-validation and sensitivity analysis demonstrate the cross-file generalization capability of the proposed method. However, feature overlap still exists for some boundary samples between classes.

In addition to the conventional classification metrics, the ordered-class metrics show that the mean absolute class error was 0.385, corresponding to a mean absolute gap error of 0.0385 mm. The adjacent-level tolerant accuracy reached 88.39%, indicating that most misclassifications occurred within a limited ordinal distance. Therefore, although local overlap still existed among some air-gap states, the severity of most prediction errors was relatively small from the perspective of ordered air-gap levels.

The difference between the last-file split and file-grouped cross-validation results indicates that the diagnostic difficulty varies among acquisition files. The last-file split evaluates the model on one selected file from each air-gap class, whereas file-grouped cross-validation repeatedly changes the held-out files and therefore provides a more stringent estimate of cross-file generalization. The lower mean performance under file-grouped cross-validation may be related to several factors, including minor file-to-file differences in brake action consistency, local mechanical adjustment states, sensor coupling condition, environmental noise, and possible temperature-related drift during repeated operation. Therefore, the file-grouped cross-validation result is regarded as the main generalization evidence in this study, while the last-file split result is used as an auxiliary hold-out evaluation.

## 5. Conclusions

This study presented an engineering-oriented sensor-based framework for elevator brake air-gap monitoring under controlled experimental conditions. The proposed RMS-IE-SVM method combines RMS-envelope-guided transient impact extraction, interpretable multi-domain feature construction, and an RBF-kernel SVM classifier. The main conclusions are as follows:

(1) The short-time RMS envelope can effectively characterize the transition between stationary and operating states of the elevator brake. By restricting peak searching to the neighborhoods of state-transition boundaries, the proposed method reduces the interference of non-target vibration stages and improves the localization of brake-release and brake-engagement impacts in long-duration vibration recordings.
(2) Brake-release impact features showed stronger sensitivity to controlled air-gap variation than brake-engagement features and combined brake-release and brake-engagement features. Using the fixed y-axis brake-release features, RMS-IE-SVM achieved an accuracy of 89.77% and a Macro F1 of 89.85% under the last-file split setting and a mean accuracy of 83.62% and a mean Macro F1 of 79.07% under file-grouped cross-validation. The ordered-class metrics further showed that the full-data file-grouped evaluation had a mean absolute gap error of 0.0385 mm and an adjacent-level tolerant accuracy of 88.39%.
(3) The parameter sensitivity analysis showed that the RMS-envelope-guided extraction process was stable within the tested local parameter range. All tested settings extracted 985 complete cycles, and the file-grouped cross-validation accuracy varied only within a narrow range from 83.42% to 83.72%. This indicates that the adopted extraction parameters were representative of a stable parameter region rather than a unique over-tuned setting.
(4) The common-sample multi-axis comparison showed that vibration direction influences air-gap recognition performance. Under the same 985 samples, 76 files, and identical grouped cross-validation folds, X + Y + Z feature-level fusion achieved the best cross-file performance, with a mean accuracy of 89.63% and a mean Macro F1 of 85.94%. This suggests that multi-axis vibration information can improve the robustness of sensor-based elevator brake air-gap identification.

It should be noted that the experiments in this study were conducted on a single traction machine, with a single sensor installation position, under no-load operation and controlled air-gap adjustment conditions. Real long-term brake-shoe wear, different loads, different running speeds, multiple equipment types, and independent non-vibration validation methods have not yet been fully covered. Future work will introduce real in-service wear samples, multi-load operating conditions, cross-equipment validation, and independent verification methods such as coil-current/voltage transient analysis, brake microswitch timing, displacement measurement, or static inspection records to further evaluate the generalization and practical applicability of the proposed framework.

## Figures and Tables

**Table 1:** Experimental equipment and main technical parameters.

Equipment	Model/Manufacturer	Main Technical Parameters or Purpose
Elevator traction machine	Shenyang Blue-Light gearless PMSM traction machine; Shenyang Blue-Light Drive Technology Co., Ltd., Shenyang, China	Rated speed: 168 r/min; rated power: 11.7 kW; rated torque: 665 N·m
Block brake	SDZK-8500; Wuhu Dazhong Electromechanical Manufacturing Co., Ltd., Wuhu, China	Rated voltage: 110 V; rated power: 140 W
Triaxial accelerometer	PCB 356A33; PCB Piezotronics, Inc., Depew, NY, USA	Sensitivity: 10 mV/g; measurement range: ±500 g pk; electrical connector: 1/4-28 4-pin
Data acquisition front end	Siemens LMS SCADAS Mobile; Siemens Digital Industries Software, Leuven, Belgium	Used for signal conditioning of the acceleration sensor, vibration signal acquisition, and data transmission
Test analysis software	Siemens LMS Test.Lab (Simcenter Testlab 2306); Siemens Digital Industries Software, Leuven, Belgium	Used for signal acquisition control, real-time monitoring, and data storage

**Table 2:** Dataset sample distribution.

Brake Air Gap	Number of Files	Brake-Release Samples	Training Samples	Test Samples
0.30 mm	12	159	145	14
0.40 mm	11	160	146	14
0.50 mm	15	169	154	15
0.60 mm	13	171	156	15
0.70 mm	13	173	158	15
0.80 mm	12	153	138	15

**Table 3:** Sensitivity analysis of RMS-envelope extraction parameters.

q	α_on_	α_off_	Complete Cycles	Last-File Acc.	Last-File Macro F1	Grouped CV Acc.	Grouped CV Macro F1	Grouped CV MACE	Adjacent Acc.
1	0.45	0.25	985	89.77%	89.85%	83.72%	79.15%	0.379	88.58%
2	0.45	0.25	985	89.77%	89.85%	83.62%	79.07%	0.385	88.39%
3	0.45	0.25	985	90.91%	90.89%	83.64%	79.13%	0.380	88.60%
2	0.35	0.25	985	90.91%	90.89%	83.55%	79.01%	0.381	88.60%
2	0.55	0.25	985	89.77%	89.85%	83.42%	78.82%	0.388	88.30%
2	0.45	0.15	985	89.77%	89.85%	83.62%	79.07%	0.385	88.39%
2	0.45	0.35	985	89.77%	89.85%	83.62%	79.07%	0.385	88.39%

**Table 4:** Correlation analysis between key brake-release impact features and brake air gap.

Feature	Pearson r	Pearson *p*	Spearman r	Spearman *p*	Abs. Spearman r
RMS	0.6758	$2.08\times 10^{-132}$	0.7333	$6.60\times 10^{-167}$	0.7333
Energy	0.6242	$1.72\times 10^{-107}$	0.7333	$6.60\times 10^{-167}$	0.7333
Log-energy	0.7101	$6.22\times 10^{-152}$	0.7333	$6.60\times 10^{-167}$	0.7333
10–20 kHz band ratio	−0.6683	$1.82\times 10^{-128}$	−0.6676	$4.32\times 10^{-128}$	0.6676
Crest factor	−0.5955	$1.46\times 10^{-95}$	−0.6521	$2.34\times 10^{-120}$	0.6521
Spectral entropy	0.6094	$3.56\times 10^{-101}$	0.6166	$3.37\times 10^{-104}$	0.6166

**Table 5:** Comparison of diagnostic performance using different impact input features.

Input Type	Best Model	Accuracy	Macro F1	Weighted F1	Number of Features
$X_{\mathrm{open}}$	Proposed RMS-IE-SVM	89.77%	89.85%	89.66%	33
$X_{\mathrm{close}}$	Logistic Regression	71.59%	71.28%	70.79%	33
$X_{\mathrm{open}\text{-}\mathrm{close}}$	Random Forest	81.82%	82.51%	82.11%	94

**Table 6:** Performance comparison of traditional machine learning models based on brake-release impact features.

Model	Accuracy	Macro Precision	Macro Recall	Macro F1	Weighted F1
Proposed RMS-IE-SVM	89.77%	90.01%	90.00%	89.85%	89.66%
Random Forest	82.95%	83.52%	83.02%	83.10%	83.04%
Extra Trees	82.95%	83.60%	83.02%	82.95%	82.89%
Logistic Regression	80.68%	80.64%	81.03%	80.49%	80.16%
KNN	70.45%	73.17%	70.56%	71.37%	71.28%

**Table 7:** Comparison of single-axis and multi-axis inputs under two cross-file evaluation settings.

Input	Features	Last-File Acc.	Last-File Macro F1	Grouped CV Acc.	Grouped CV Macro F1	MAGE (mm)	Adjacent Acc.
*X*-axis	33	84.09%	84.22%	86.52%	82.66%	0.0355	88.73%
*Y*-axis	33	89.77%	89.85%	83.62%	79.07%	0.0385	88.39%
*Z*-axis	33	87.50%	87.58%	87.41%	83.86%	0.0337	89.43%
Vector magnitude	33	87.50%	87.61%	82.31%	77.78%	0.0399	88.34%
X + Y + Z feature fusion	99	89.77%	89.83%	89.63%	85.94%	0.0276	91.44%

Note: MAGE and Adjacent Acc. are calculated from the file-grouped cross-validation predictions.

**Table 8:** Results of cross-file validation and ordered-class evaluation.

Data Setting	Evaluation	Accuracy	Macro F1	MACE	MAGE (mm)	Adjacent-Level Tolerant Accuracy	Samples	Files
Full data	File-grouped CV	83.62%	79.07%	0.385	0.0385	88.39%	985	76
Without suspect files	File-grouped CV	83.65%	80.23%	0.381	0.0381	88.45%	955	74
Full data	Last-file split	89.77%	89.85%	0.261	0.0261	90.91%	985	76
Without suspect files	Last-file split	88.89%	87.33%	0.296	0.0296	90.12%	955	74

## Data Availability

The original contributions presented in this study are included in the article. Further inquiries can be directed to the corresponding author.
